# Prediction of Bond Strength in Corroded Reinforced Concrete Using SVM and XGB Methods

**DOI:** 10.3390/ma19101928

**Published:** 2026-05-08

**Authors:** Zhi-Qiang Chen, Zhuang Chen, Ying-Zi Zhong

**Affiliations:** 1 School of Architecture and Civil Engineering, Chengdu University, Chengdu 610106, China; 2025210097@my.swjtu.edu.cn (Z.-Q.C.); chenpostg@gmail.com (Z.C.); 2 China Railway Eryuan Engineering Group Co., Ltd., Chengdu 610031, China; 3 School of Civil Engineering, Southwest Jiaotong University, Chengdu 610031, China

**Keywords:** support vector machine, extreme gradient boosting, corroded reinforced concrete, bond strength, SHAP method

## Abstract

The bond strength of corroded reinforced concrete (CRC) structures is critical for structural safety and long-term durability. However, the corrosion-induced bond degradation process is influenced by multiple, coupled factors and exhibits complex, nonlinear behavior, making it difficult for traditional theoretical models to provide accurate predictions. To address this challenge, this study proposes a novel, unified prediction framework based on machine learning techniques. A total of 391 experimental datasets were collected and compiled, covering key parameters including bond strength, reinforcing bar diameter, yield strength, concrete cover thickness, concrete compressive strength, mass loss rate due to corrosion, and the presence of stirrups. Support Vector Machine (SVM) and Extreme Gradient Boosting (XGBoost) algorithms were employed to develop predictive models for bond strength. Model training and testing were performed using 10-fold cross-validation. Furthermore, the SHapley Additive exPlanations (SHAP) approach was introduced to enhance model interpretability and quantitatively assess the influence of each input feature, revealing that mass loss rate and bar diameter are the dominant factors. This study effectively bridges the research gap between high-precision black-box algorithms and the need for physical interpretability in engineering. The results demonstrate that (1) the proposed XGBoost model significantly outperforms traditional empirical formulations, achieving a high coefficient of determination (R^2^ = 0.893) and a much lower coefficient of variation (25.85%) on the testing set, and (2) the SHAP analysis reveals that the machine learning predictions are highly consistent with established physical mechanisms, successfully capturing the negative impact of splitting tensile stresses caused by rust expansion and the positive confinement effect of stirrups. Overall, the proposed models demonstrate superior accuracy, robustness, and generalization capability, providing an effective tool and theoretical basis for evaluating bond behavior and designing durable CRC structures with broad engineering applicability.

## 1. Introduction

In corroded reinforced concrete structures, the bond strength between steel reinforcement and concrete is one of the key parameters determining the overall mechanical performance and durability of the structure [1]. With increasing service time, corrosive agents—such as chloride salts and marine aerosols—gradually penetrate and attack the reinforcement, leading to the development of interfacial microcracks and transverse expansion. These processes cause a progressive degradation of bond performance, significantly weakening the composite action between steel and concrete and ultimately reducing the structural load-bearing capacity and service life [2,3,4]. This issue is particularly critical in marine environments or regions exposed to de-icing salts.

The degradation of bond strength is a highly complex and nonlinear process governed by the coupled effects of multiple factors, including the degree of steel corrosion, concrete strength, concrete cover thickness, rebar diameter, and steel yield strength [5,6]. In recent years, extensive experimental and numerical studies have been conducted to elucidate the mechanisms of corrosion-induced deterioration at the steel-concrete interface. Lee et al. [7] combined accelerated corrosion pull-out tests with finite element analysis and found that both bond strength and stiffness exhibit a pronounced decrease with increasing corrosion levels. They further proposed predictive equations suitable for numerical simulations. Lundgren [8], based on a three-dimensional finite element model, revealed that splitting stresses induced by the volumetric expansion of corrosion products constitute a key mechanism for bond degradation. Lin et al. [9] demonstrated an exponential decay relationship between surface crack width and bond strength caused by corrosion and developed a practical prediction model considering the confining effect of stirrups. In a subsequent study, Lin et al. [10] reported that the simultaneous corrosion of longitudinal reinforcement and stirrups significantly exacerbates bond deterioration, and they proposed a bond-slip model accounting for the dual corrosion effect. Furthermore, recent research has indicated that chloride-induced stirrup corrosion can fundamentally alter the failure pattern of RC members and lead to severe degradation in shear behavior [11], highlighting the critical role of stirrups in the durability assessment of corroded structures. Yalciner and Kumbasaroglu [12] systematically investigated the influence of concrete strength, axial load ratio, and corrosion level on bond-slip behavior under cyclic loading and developed an empirical model applicable to corroded reinforced concrete columns. Feng et al. [13], from the perspective of construction techniques, found that different rust-removal methods have markedly different effects on restoring the bond performance of corroded steel bars, with mechanical grinding proving the most effective for retaining bond strength. Zheng et al. [14] reported that mild corrosion may slightly enhance bond strength due to increased surface roughness, whereas severe corrosion substantially weakens interfacial performance. They proposed an improved bond-slip constitutive model accordingly. Moreover, Zheng et al. [15] pointed out that most existing models are based on idealized assumptions and fail to fully capture the complex, multi-factor coupling behavior observed in real structures. Li et al. [16] developed an analytical model for corroded steel strands embedded in concrete, which effectively predicts bond performance across various corrosion levels. Overall, while many studies have focused on developing theoretically derived empirical models, these models often struggle to accurately capture the interactive effects among multiple influencing variables, thereby limiting their applicability in complex engineering conditions [17].

In recent years, machine learning techniques have been increasingly introduced into structural engineering and materials science due to their strong capability in handling high-dimensional and nonlinear problems, ranging from the shear strength prediction of RC columns to material property evaluation, providing an effective means to overcome the limitations of traditional empirical and theoretical models [18]. Recent research in Structural Engineering International has also demonstrated the efficacy of these methods; Mohan et al. [19] utilized SVM for damage classification, while Singh et al. [20] applied data-driven models for seismic assessment. Among various ML algorithms, Support Vector Machine and Extreme Gradient Boosting have shown excellent performance in high-dimensional data fitting and feature selection, demonstrating superior stability and predictive accuracy [21,22,23,24,25]. Specifically, recent studies have highlighted the superior capability of XGBoost in accurately predicting the nonlinear structural parameters of rectangular RC columns [26], further validating its applicability in complex concrete engineering problems. Several scholars have applied machine learning approaches to predict interfacial properties of concrete materials. For instance, Chen et al. [27] developed a predictive model for the bond strength between fiber-reinforced polymer and concrete using an ensemble learning approach. Nguyen et al. [28] employed the XGBoost algorithm to construct a predictive model for the compressive strength of ultra-high-performance concrete, achieving higher accuracy compared to conventional empirical formulations. Li et al. [29] proposed an XGBoost-based model for predicting the bond-slip relationship of reinforced concrete using a comprehensive dataset of 1056 experimental samples. Although these studies have made notable progress, most of them primarily focus on uncorroded reinforcement or FRP-concrete interfaces. Systematic predictive models specifically addressing the bond performance of corroded reinforced concrete remain scarce. Although advanced deep learning models have achieved remarkable success in automated defect detection [30], these approaches often function as ‘black boxes’. Existing predictive models for bond strength similarly lack transparent interpretability of their internal mechanisms, which limits their applicability for practical engineering diagnosis.

To address the existing research gaps, the primary objective of this research is to develop a unified and high-precision machine learning framework for predicting the bond strength of corroded reinforced concrete structures, thereby overcoming the limitations of traditional empirical models in handling complex, nonlinear degradation behaviors. Furthermore, the new findings of this study are two-fold: (1) the proposed XGBoost model demonstrates remarkable superiority over traditional formulations, achieving significantly higher prediction accuracy and robustness; (2) by successfully coupling the machine learning algorithm with the SHAP method, this study effectively breaks through the “black-box” limitation, providing mechanics-consistent explanations that align with fundamental physical laws. The research utilizes a database comprising 391 experimental samples, where six key input features—mass loss rate of reinforcement, bar diameter, yield strength, concrete cover thickness, concrete compressive strength, and the presence or absence of stirrups—are adopted to predict bond strength. A series of predictive models were developed and systematically compared to evaluate the performance and accuracy of the SVM and XGBoost algorithms. To enhance model interpretability, the SHAP method was incorporated to quantitatively reveal the contribution and influence trends of each input parameter on the predicted bond strength. Moreover, the proposed models were benchmarked against three classical theoretical formulations to comprehensively assess their predictive accuracy, robustness, and generalization capability. The findings of this study are expected to provide both theoretical insights and practical tools for the performance assessment and strengthening optimization of corroded reinforced concrete structures.

## 2. Experimental Dataset

**Figure 1:**
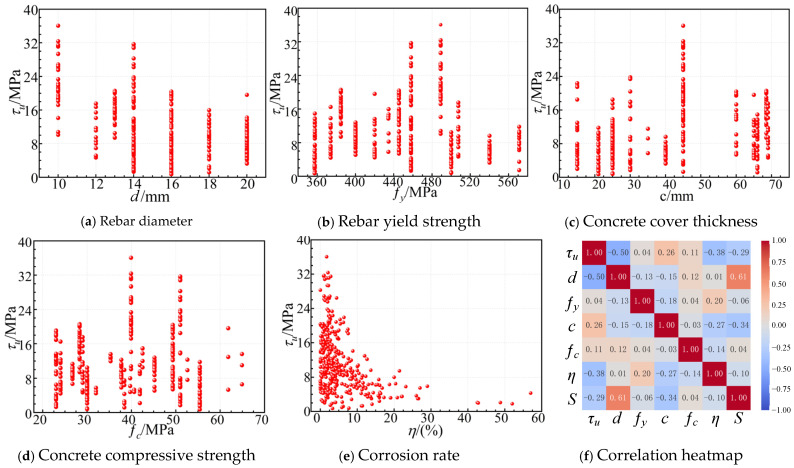
Analysis of dataset variables. (**a**–**e**) Distribution of input and output parameters in the compiled dataset; (**f**) Pearson correlation matrix of all variables.

To develop a machine learning model capable of predicting bond strength, this study compiled and integrated experimental data on corroded reinforced concrete from the existing literature [5,31,32,33,34,35,36,37,38,39,40,41,42], resulting in a dataset containing 391 valid samples. The dataset covers the primary factors influencing the bond performance between steel and concrete, ensuring good representativeness and applicability. Specifically, the 391 data samples collected from 13 independent and representative studies cover a broad spectrum of critical engineering parameters, such as concrete strengths from 23.0 to 64.9 MPa and mass loss rates up to 57.0%, reflecting diverse real-world conditions. The distributions of input and output parameters are presented in Figure 1 and Table 1. The dataset includes six input variables: bar diameter d, yield strength of reinforcement $f_{y}$, concrete cover thickness c, concrete compressive strength $f_{c}$, mass loss rate of steel bars η, and the presence of stirrups (0 = without, 1 = with, as a binary categorical variable, denoted as S in subsequent figures). The output variable is the bond strength $\tau_{u}$ at the steel-concrete interface, ranging from 0.704 MPa to 36.070 MPa. The ultimate bond strength is defined as the maximum average bond stress along the embedded length, calculated as the peak pull-out force divided by the surface area of the bonded reinforcement. All data were obtained from conventional pull-out or modified pull-out tests, where a monotonic tensile load was applied until bond failure occurred. The original references were carefully screened to ensure the comparability of experimental conditions and the consistency of data. This dataset provides a solid foundation for building a high-accuracy, strong generalization machine learning model for bond strength prediction.

While the fundamental causes of reinforcement corrosion (e.g., chloride attack and carbonation) significantly govern the initiation phase, this study focuses specifically on evaluating the residual bond strength after corrosion has progressed to a measurable state. Consequently, the mass loss rate is employed as a comprehensive macroscopic indicator of the corrosion level. Furthermore, it is noted that environmental parameters such as internal moisture content are rarely consistently reported in the existing experimental literature, precluding their inclusion as input variables in the current dataset. Addressing these environmental factors remains a critical objective for future high-fidelity data-driven research.

To gain a deeper understanding of the interactions among variables and their potential impact on the model’s predictive performance, this study performs component distribution and correlation analysis on the constructed dataset. Pearson’s correlation coefficient method is used to assess the linear correlations between input variables and their relationship with the output variable (interface bond strength), and a correlation matrix is presented. The analysis results show that the absolute values of the Pearson correlation coefficients between input variables range from −0.34 to 0.61, with no significant multicollinearity issues detected. Specifically, the correlations between variables such as rebar diameter, concrete compressive strength, cover thickness, and mass loss rate are all below the empirical threshold of |r| = 0.7, indicating that they can serve as independent features in model training. Additionally, the correlations between the input variables and bond strength range from −0.50 to 0.26, with no significant linear correlation observed. This further suggests that the factors influencing bond strength exhibit a complex, nonlinear mechanism, making them suitable for modeling with machine learning techniques. Therefore, all selected variables can be considered valid input features for model construction without negatively impacting the model’s predictive performance or interpretability due to variable redundancy.

## 3. Machine Learning Algorithms

To address the inherent high nonlinearity and coupling effects among the factors influencing bond strength, this study employed two representative machine learning algorithms—namely, SVM, a single-learner model, and XGBoost, an ensemble method—to construct a stable and reliable prediction framework.

### 3.1. Support Vector Machine

Support Vector Machine, initially proposed by Vapnik et al. [43], is based on statistical learning theory and has been widely applied to classification and regression problems. Support Vector Regression (SVR) is an extension of SVM specifically designed for solving regression tasks. Similarly to SVM in classification, SVR also aims to find an optimal hyperplane. However, in regression, its goal is to find a function that approximates the target output as closely as possible while ensuring the model retains good generalization ability.

The basic idea of SVR is to allow a certain prediction error (determined by a threshold ε), but the number of data points with errors exceeding the ε margin should be minimized. The core concept is to strike a balance between model complexity and prediction accuracy. Given a training set $\left\{\left(x_{1},y_{1}\right),\{\left(x_{2},y_{2}\right),\ldots ,\left(x_{n},y_{n}\right)\right\}$, where $x_{i}\in R^{d}$ represents the input features and $y_{i}\;\in R$ represents the target values, SVR seeks a function *f*(*x*) such that it satisfies the following condition for all samples:

(1) $$\left|f\left(x_{i}\right)-y_{i}\right|\leq \varepsilon$$

Within the *ε*-approximation error margin, the goal is to minimize the model complexity. The optimal function is typically represented as

(2) $$f\left(x\right)=\omega^{T}\cdot \phi \left(x\right)+b$$

where ω is the weight vector, $\phi \left(x\right)$ is the kernel function mapping, and b is the bias term.

To optimize the model, we introduce slack variables $\xi_{i}$ and $\xi_{i}^{*}$ to tolerate errors exceeding ε, and the problem is addressed by the following optimization objective:

(3) $$\min\left[R\left(\omega ,\xi_{i},\xi_{i}^{*}\right)\right]=\frac{1}{2}{\left\|\omega \right\|}^{2}+C\sum \left(\xi_{i}+\xi_{i}^{*}\right)$$

The constraint conditions are

(4) $$y_{i}-\left(\omega^{T}\cdot \phi \left(x_{i}\right)+b\right)\leq \varepsilon +\xi_{i}$$

(5) $$\left(\omega^{T}\cdot \phi \left(x_{i}\right)+b\right)-y_{i}\leq \varepsilon +\xi_{i}^{*}$$

(6) $$\xi_{i},\xi_{i}^{*}\geq 0$$

where C > 0 is the regularization parameter, which controls the trade-off between model complexity and training error.

By applying the Lagrangian duality theory, the dual problem can be obtained. The solution method uses the kernel function to replace the explicit computation of $\phi \left(x\right)$:

(7) $$K\left(x_{i},x_{j}\right)={\;\phi \left(x_{i}\right)}^{T}\phi \left(x_{j}\right)$$

The final optimization problem is transformed into the calculation of the weight coefficients of the support vectors, resulting in the prediction function:

(8) $$f\left(x\right)=\sum_{i=1}^{n}\left(\alpha_{i}-\alpha_{i}^{*}\right)K\left(x_{i},x\right)+b$$

where $\alpha_{i}$ and $\alpha_{i}^{*}$ are the Lagrange multipliers.

In this study, the SVR model employs the Radial Basis Function kernel, and the optimal parameter combination is determined through preliminary experiments: ε = 0.01, C = 4, and γ = 0.8, with all other parameters set to their default values. The model demonstrates good performance in fitting the nonlinear trends of bond strength and serves as a benchmark for comparison with the ensemble algorithm.

### 3.2. Extreme Gradient Boosting Tree

To improve the accuracy of fitting the nonlinear relationship of bond strength, this paper introduces the XGBoost algorithm as an ensemble learning method. Proposed by Tianqi Chen et al. [44] in 2016, XGBoost is an enhancement of the traditional Gradient Boosting Decision Tree. Its core idea is to iteratively build multiple decision trees, fitting the residuals from the previous model, thereby progressively optimizing overall predictive performance. Additionally, XGBoost incorporates L1 and L2 regularization terms to mitigate overfitting and utilizes approximation algorithms and parallel processing to enhance training efficiency.

In this study, the main hyperparameters of the XGBoost model are set as follows: the number of trees is 148, the learning rate is 0.2, the maximum tree depth is 3, the minimum sum of instance weight for a child node is 3, both the subsample ratio for training samples and the feature subsample ratio are 0.7, and the minimum loss reduction is set to 0.5. The remaining parameters are set to their default values. These parameters were optimized using cross-validation and grid search methods to enhance the model’s generalization ability in predicting bond strength.

The XGBoost predictive model is composed of multiple regression trees. Let the training set be $\left\{\left(x_{1},y_{1}\right),\ldots ,\left(x_{n},y_{n}\right)\right\}$, where $x\in R^{n\times m}$ represents the *m* features (influencing factors), and $y_{i}$ is the bond strength $\left(y\in R^{n\times 1}\right)$. The final prediction of the model is the sum of the predictions from all trees:

(9) $$\hat{y_{i}}=\sum_{k=1}^{k}f_{k}\left(x_{i}\right)\;\;\;\;\;\;\;f_{k}\in F$$

where F represents the set of regression trees, k is the number of classification and regression trees, and $f_{k}\left(x_{i}\right)$ is the prediction of the *k*-th regression tree. The XGBoost algorithm aims to render the predicted values $y_{i}$, and the objective function is expressed as

(10) $$Obj=\sum_{i=1}^{n}L(y_{i},\hat{y_{i}})+\sum_{k=1}^{K}\Omega \left(f_{k}\right)$$

where $L(y_{i},\hat{y_{i}})$ is the loss function used to evaluate the accuracy of the predicted values $\hat{y_{i}}$, and $\Omega (f_{k})$ represents the regularization term to prevent overfitting. Let $\hat{y_{i}^{k}}$ be the prediction after the *k*-th iteration; the objective function can be expressed as

(11) $${Obj}^{k}=\sum_{i=1}^{n}L\left(y_{i},{\hat{y}}_{i}^{k-1}+f_{k}\left(x_{i}\right)\right)+\Omega \left(f_{k}\right)+C$$

In the equation, C is a constant. After performing a Taylor expansion of the loss function and removing terms that are independent of $f_{k}$, the equation can be simplified to

(12) $$g_{i}=l^{\prime }\left(y_{i},{\hat{y}}_{i}^{k-1}\right)$$

(13) $$h_{i}=l^{\prime \prime }\left(y_{i},{\hat{y}}_{i}^{k-1}\right)$$

(14) $${Obj}^{k}=\sum_{i=1}^{n}\left(g_{i}f_{k}\left(x_{i}\right)+\frac{1}{2}h_{i}f_{k}^{2}\left(x_{i}\right)\right)+\Omega \left(f_{k}\right)$$

If $f_{k}$ contains *L* leaves, $I_{j}$ is the sample set of leaf *j*, and *w*_*j*_ is the weight of leaf *j*, then $\Omega \left(f_{k}\right)$ can be expressed as

(15) $$\Omega \left(f_{k}\right)=\lambda L+\frac{1}{2}\mu \sum_{j=1}^{L}w_{j}^{2}$$

In the equation, λ and μ are constants. Therefore, the equation can be simplified to

(16) $$Obj=\sum_{j=1}^{L}\left[G_{i}w_{j}+\frac{1}{2}\left(H_{i}+\mu \right)w_{j}^{2}\right]+\lambda L$$

where $G_{i}=\sum_{i\in I_{j}}g_{i}$, $H_{i}=\sum_{i\in I_{j}}h_{i}$. From the equation, it can be observed that $G_{i}w_{j}+\frac{1}{2}(H_{i}+\mu )w_{j}^{2}$ is a quadratic function with respect to $w_{j}$. Therefore, the optimal value of the objective function can be expressed as

(17) $$Obj=-\frac{1}{2}\sum_{j=1}^{L}\left(\frac{G_{j}^{2}}{H_{j}+\mu }\right)+\lambda L$$

## 4. Model Implementation and Result Analysis

### 4.1. Model Training and Testing Procedure

This study establishes a machine learning-based modeling framework for predicting the bond strength of corroded reinforced concrete. The overall workflow consists of six major stages: data collection, data preprocessing, model training, hyperparameter optimization, model validation, and model interpretation. The detailed procedure is as follows:

Step 1—Data Collection: Experimental data related to the bond strength of corroded reinforced concrete were systematically gathered and organized from the existing literature. A dataset containing 391 samples was constructed for subsequent model training and evaluation.

Step 2—Data Preprocessing: Prior to normalization, an adequate data cleaning process was performed to handle potential outliers. Statistical methods, such as the interquartile range (IQR) technique, were employed to identify and exclude extreme anomalous values caused by potential measurement errors, ensuring the reliability of the dataset. Subsequently, all input features and output variables were normalized using the min–max normalization method, mapping the values to the [0, 1] range to eliminate the effect of dimensionality. The data was then randomly split into a training set (312 samples, 80%) and a testing set (79 samples, 20%).

Step 3—Model Training: Based on the training dataset, two machine learning models—SVM and XGBoost—were developed using Python (version 3.11.5). Specifically, the SVM model was implemented via the scikit-learn library (version 1.6.1), and the XGBoost model was constructed using the XGBoost package (version 2.1.4). Multiple performance metrics were employed to conduct a preliminary evaluation of model learning capacity and stability.

Step 4—Hyperparameter Optimization: A grid search combined with k-fold cross-validation was employed to systematically optimize key model hyperparameters, aiming to enhance the model’s generalization ability and prediction accuracy.

Step 5—Model Validation: The trained models were evaluated on the independent testing dataset. Performance metrics included the coefficient of determination (R^2^), root mean square error (RMSE), mean absolute error (MAE), and mean absolute percentage error (MAPE). The predictive performance of different models was comprehensively analyzed and compared.

**Figure 2:**
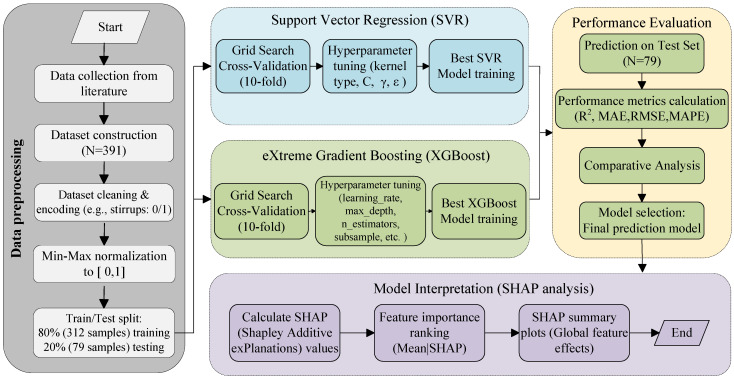
Machine learning framework for predicting bond strength of corroded reinforced concrete.

Step 6—Model Interpretation: To enhance the model’s interpretability, the SHAP method was introduced. This method provides both global and local explanations of the model’s output, revealing the impact mechanisms of each input feature on the bond strength prediction and addressing the “black-box” nature of machine learning models. The overall research framework of this study is illustrated in Figure 2.

### 4.2. Evaluation Metrics

To systematically evaluate the performance of the constructed machine learning models in predicting bond strength, four commonly used evaluation metrics were selected: R^2^, RMSE, MAE, and MAPE. Specifically, R^2^ is used to measure the model’s ability to explain the variance in the data, RMSE and MAE reflect the deviation between the model’s predicted values and the actual values, and MAPE provides a relative error measure. The mathematical definitions of these metrics are shown in Table 2.

### 4.3. Model Predictive Performance and Validation Analysis

Table 3 presents the evaluation metrics—R^2^, RMSE, MAE, and MAPE—of both the SVM and XGBoost models on the training and testing sets. On the training set, the R^2^ value of the XGBoost model is 0.9460, significantly higher than the 0.8531 of the SVM model, indicating that XGBoost fits the training data more effectively. Meanwhile, the RMSE for the XGBoost model decreases from 2.5940 to 1.6125 (a relative reduction of approximately 37.84%), the MAE decreases from 1.7703 to 1.1731 (a reduction of about 33.73%), and the MAPE decreases from 24.9984 to 15.4347 (a reduction of approximately 38.27%), all indicating superior fitting performance and error control capabilities. On the testing set, XGBoost also outperforms SVM. Its R^2^ value is 0.8934, higher than SVM’s 0.8593. Additionally, the RMSE decreases from 2.8182 to 2.2373 (a relative reduction of approximately 20.61%), the MAE drops from 2.1075 to 1.6802 (a reduction of about 20.28%), and the MAPE decreases from 24.3626 to 17.8607 (a reduction of approximately 26.69%). These results show that the XGBoost model demonstrates stronger generalization ability and stability during both the training and prediction phases. Overall, XGBoost outperforms the SVM model across multiple evaluation metrics, showcasing more robust and accurate bond strength prediction performance. A detailed statistical analysis of the relative prediction ratios ($\tau_{real}/\tau_{pred}$) is further discussed in Section 5. Furthermore, the proposed models are highly scalable and can be continuously updated by simply appending new experimental datasets to the training matrix.

**Figure 3:**
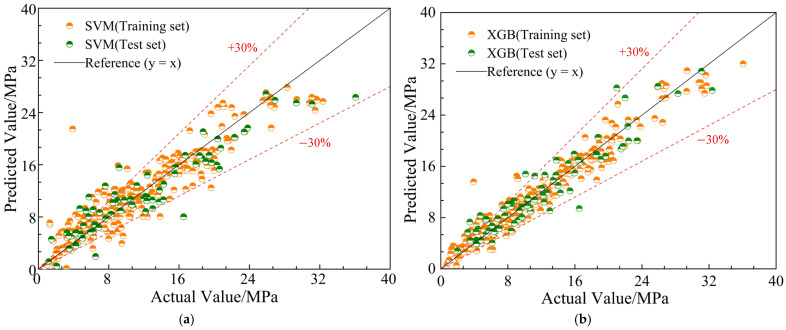
Fitting results of bond strength for ML models. (**a**) SVM model. (**b**) XGB model.

Figure 3 shows the regression scatter plots of the SVM and XGBoost models in predicting bond strength. To assess the degree of fit between the model’s predicted values and the actual values, diagonal lines and auxiliary lines at ±30% error limits are plotted. It can be observed that, compared to the SVM model, the predictions of the XGBoost model are more tightly distributed within the ±30% error margin, with a higher proportion of points falling within this range. This indicates that the XGBoost model provides more accurate predictions of bond strength, with stronger error control and better sample fitting performance. The relatively dense scatter plot further validates the adaptability advantage of the XGBoost algorithm in handling nonlinear multivariable relationships. 

**Figure 4:**
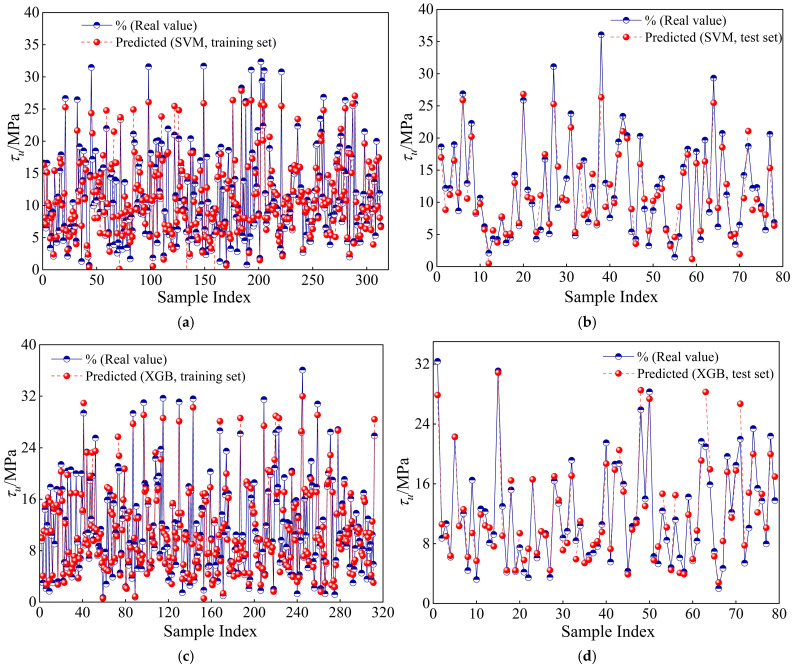
Comparison between actual and predicted results from ML models. (**a**) SVM: training set. (**b**) SVM: test set. (**c**) XGB: training set. (**d**) XGB: test set.

Figure 4 compares the performance of the SVM and XGBoost models in predicting bond strength in corroded reinforced concrete. It shows the relationship between predicted values and experimentally measured values (see Figure 4). From the figure, it is evident that both models exhibit a good fit with the experimental data, indicating their feasibility for this problem. Further observation reveals that the XGB model’s predicted values are more closely aligned with the diagonal line, with smaller fluctuations in error, suggesting higher accuracy and stability. This result further validates the advantage of ensemble learning methods in addressing high-dimensional nonlinear regression problems. In conclusion, the XGB model demonstrates superior predictive performance and generalization ability compared to traditional single algorithms such as SVM, making it more suitable for modeling CRC bond strength in complex environments.

### 4.4. Model Interpretation

To enhance the interpretability of the constructed machine learning model, this study introduces the SHAP method [45]. SHAP is a game-theoretic approach that calculates the contribution of each feature to the model prediction. Specifically, the SHAP value $\phi_{i}$ for feature i is defined as the weighted average of its marginal contributions over all possible feature subsets:

(18) $$\phi_{i}=\sum_{S\subseteq N\setminus \{i\}}\frac{\left|S\right|!\left(M-\left|S\right|-1\right)!}{M!}\left[f\left(S\cup \{i\}\right)-f\left(S\right)\right]$$

where N denotes the set of all input features, M is the total number of input features, and S denotes any subset of features that excludes feature i. The term $f\left(S\cup \{i\}\right)-f\left(S\right)$ represents the marginal contribution of feature i to the model prediction.

For a specific prediction, the SHAP value indicates the contribution of each feature relative to the expected model output E[f(x)]. A larger absolute SHAP value implies a stronger influence on the prediction, while a value close to zero indicates a negligible effect. Moreover, the sign of the SHAP value reflects the direction of influence: a positive value increases the predicted bond strength, whereas a negative value decreases it. In this study, the Tree SHAP algorithm was employed to efficiently compute SHAP values for the XGBoost model, facilitating the interpretation of the factors influencing bond strength.

**Figure 5:**
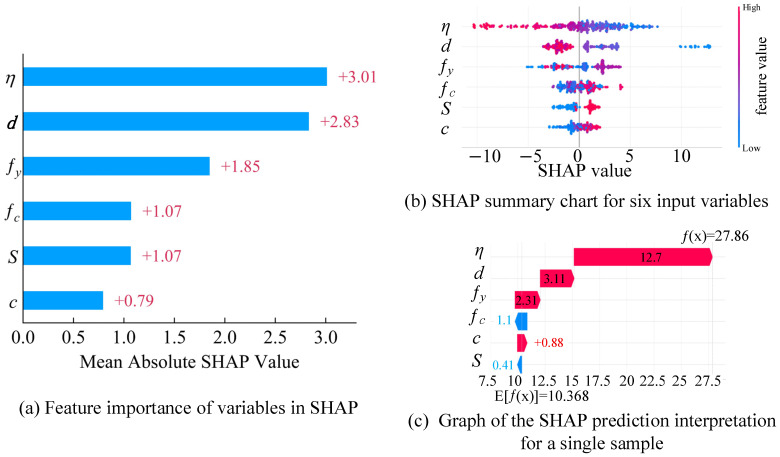
SHAP-based feature importance and interpretability analysis of the model.

In terms of global explanation, Figure 5a,b present the feature importance ranking and the distribution of SHAP values for each feature. The results indicate that the mass corrosion rate and rebar diameter are the two features contributing the most to the model’s output, highlighting their significant impact on the bond performance of corroded reinforced concrete. In contrast, the SHAP value of concrete cover thickness is relatively low, suggesting that it has a smaller weight in the model and a limited effect on the prediction outcome. Additionally, the SHAP distribution plot in Figure 5b reveals the influence trends: as the mass corrosion rate increases, its SHAP value becomes more negative, significantly lowering the predicted bond strength. From a mechanical perspective, this is because the volumetric expansion of corrosion products induces splitting tensile stresses in the concrete cover, leading to longitudinal cracking. This severe cracking deteriorates the confinement and substantially weakens the mechanical interlocking between the rebar ribs and the surrounding concrete. On the other hand, factors such as rebar strength, concrete strength, and the presence of stirrups have a positive effect on the predicted values. Physically, higher concrete strength provides a stronger gripping force, while the presence of stirrups offers effective transverse confinement, restraining the development of splitting cracks and thereby enhancing the interfacial bond capacity. These findings demonstrate that the XGBoost model not only performs numerical fitting but also accurately captures the underlying physical and mechanical behavior of the corrosion-bond phenomenon.

In terms of local explanation, Figure 5c presents the SHAP visualization analysis for a representative sample. The predicted bond strength for this sample is 27.86 MPa (with an actual value of 32.37 MPa), while the baseline value (i.e., the average predicted value across all samples) is 10.368 MPa. From the visualization, it is evident that the prediction is primarily influenced by the positive effects of rebar diameter, mass corrosion rate, rebar yield strength, and cover thickness, whereas concrete compressive strength and the absence of stirrups contribute negatively to the prediction. These results suggest that the model not only demonstrates strong predictive capability but also provides clear causal explanations at the sample level. This further validates the scientific and reliable use of machine learning methods for predicting bond strength in corroded reinforced concrete.

## 5. Comparison of Model Performance

To validate the effectiveness of the proposed model, the predictions of the XGB and SVM algorithms are compared with three existing empirical formulas. Table 4 presents the statistical comparison of the ratios between predicted bond strength and measured values, including the maximum, minimum, mean, standard deviation, and coefficient of variation. From Table 4, it can be seen that the coefficient of variation for the XGB model is 25.85%, and for the SVM model, it is 48.93%, both of which are significantly lower than those of the three empirical models (Bhargava et al. [46]: 58.13%; Kivell [47]: 61.15%; Lee et al. [7]: 65.29%), indicating that the proposed machine learning models have a significant advantage in prediction accuracy and result stability. Moreover, the mean predicted ratio for the XGB model is closest to 1.0 (1.0524), further suggesting stronger fitting capability.

Table 5 presents a comparison of the models’ regression performance evaluation metrics, including the coefficient of determination, root mean square error, mean absolute error, and mean absolute percentage error. The results show that the R^2^; values for the XGB and SVM models are 0.9354 and 0.8543, respectively, which are significantly higher than the highest R^2^; for the empirical models (Kivell model: 0.7475), demonstrating superior fitting performance.

In terms of error metrics, the XGB model achieves the lowest values for RMSE, MAE, and MAPE, with values of 1.7388, 1.2755, and 15.92, respectively. Compared to the Kivell model, the XGB model reduces RMSE by approximately 50.1%, MAE by 51.5%, and MAPE by 59.1%. The SVM model also outperforms the empirical models in these metrics, demonstrating excellent prediction accuracy and robustness.

In conclusion, the machine learning models proposed in this study—especially the XGB model—show significantly better performance in terms of prediction accuracy, error control, and stability compared to existing theoretical formulas. This highlights the superior performance and engineering application potential of these models for predicting bond strength in corroded reinforced concrete.

**Figure 6:**
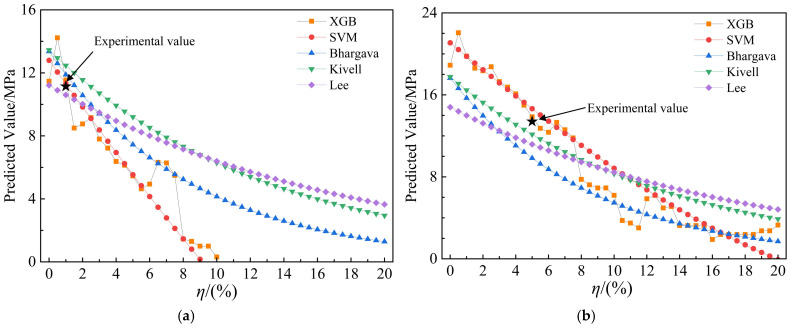
Sensitivity analysis of different models with respect to corrosion rate (**a**) Case 1; (**b**) Case 2.

To evaluate the response of different models to variations in corrosion rate (*η*), a comparative analysis was conducted between the proposed machine learning models and three classical empirical formulations, as illustrated in Figure 6a,b.

The results show that all models predict a general decreasing trend in bond strength with increasing corrosion rate, which agrees with the physical mechanism of corrosion-induced bond degradation. However, distinct differences exist in the degree of sensitivity among models. The empirical formulations exhibit relatively smooth and less steep declines, indicating lower responsiveness to corrosion variation, whereas the machine learning models—particularly XGB—demonstrate more pronounced nonlinear decreases that align more closely with the experimental observations.

To understand these distinct sensitivities, it is essential to analyze the specific boundary conditions of the two cases. These two cases represent distinct experimental specimens with different geometric and material properties. Specifically, Case 1 features a smaller rebar diameter (d=12 mm) and a particularly thick concrete cover (c=69 mm) with a compressive strength of $f_{c}=29$ MPa. In contrast, Case 2 represents a more standard configuration with d=14 mm, c=45 mm, and $f_{c}=23$ MPa. The divergence in model predictions at higher corrosion rates in Case 1 highlights a limitation of traditional empirical models: they struggle to accurately capture the sharp, nonlinear bond degradation caused by splitting failures in members with exceptionally large cover thicknesses. In Case 2, where the parameters are closer to the average configurations typically used to calibrate empirical formulas, the differences between the machine learning predictions and empirical models are notably smaller.

Overall, the XGB model shows the highest sensitivity to corrosion rate variation, with predictions most consistent with experimental results, highlighting its superior accuracy, generalization ability, and robustness in representing the complex, nonlinear corrosion–bond relationship under extreme and varied boundary conditions.

## 6. Conclusions

This study proposes a unified machine learning prediction framework based on multi-algorithm comparison, employing the Support Vector Regression and XGBoost algorithms, for predicting the bond strength of corroded reinforced concrete structures. Furthermore, the SHAP method is introduced to provide interpretability analysis of the model results. Based on 391 experimental datasets, the bond strength prediction models were constructed and evaluated for their accuracy and stability. The main conclusions of this study are summarized as follows:

1. The two constructed machine learning models are both effective in predicting the bond strength of corroded reinforced concrete. Between the two, the ensemble model, XGB, performs the best, achieving higher R^2^; values and lower error metrics on both the training and testing sets, demonstrating superior predictive accuracy and robustness.
2. By utilizing the SHAP method for both global and local explanations of the model output, key influencing factors were identified, with mass corrosion rate and rebar diameter being the primary contributors. Rebar yield strength, concrete compressive strength, and the presence of stirrups also show significant positive contributions, while mass corrosion rate negatively affects bond strength.
3. Compared to three traditional empirical formulas—Bhargava, Kivell, and Lee—the proposed machine learning models show significant advantages in terms of prediction accuracy and stability. The coefficient of variation for the predicted-to-measured value ratio of the XGB model is 25.85%, significantly lower than those of the SVM model (48.93%) and the empirical models (CV > 58.13%), indicating higher consistency and better engineering applicability.
4. This study carries out a successful coupling of XGBoost and SHAP to provide not only high-accuracy predictions but also quantitative, mechanics-consistent explanations for the bond degradation phenomenon. This fills the crucial research gap between predictive capability and physical interpretability in applying ML to civil engineering. For future work, the model can be further enriched by incorporating additional environmental parameters, such as concrete moisture content, and by utilizing in situ testing data to continuously train and optimize the algorithm for broader practical applications.

In summary, the hybrid machine learning prediction framework based on SHAP analysis proposed in this study not only improves the prediction accuracy for bond strength of corroded reinforced concrete but also provides interpretability of the model’s internal mechanisms. It demonstrates strong potential for broader application and value in engineering practice.

## Figures and Tables

**Table 1:** Input and output parameters statistics.

Classification	Variables	Data			
		Min	Max	Mean	SD
Input	d/mm	10	20	15.79	3.17
Input	$f_{y}/\mathrm{MPa}$	357.23	571.06	441.97	57.49
Input	c/mm	15	69	40.64	17.85
Input	$f_{c}/\mathrm{MPa}$	23.0	64.9	37.69	11.02
Input	η/(%)	0.0	57.0	5.46	7.27
Input	Stirrup Presence (0/1)	0	1	0.50	0.50
Output	$\tau_{u}/\mathrm{MPa}$	0.704	36.070	11.36	6.94

**Table 2:** Statistical indicators.

Metric	Formula	Ideal Value
$R^{2}$	$R^{2}=\frac{\sum_{i=1}^{n}{\left(x_{i}-p_{i}\right)}^{2}-\sum_{i=1}^{n}{\left(x_{i}-y_{i}\right)}^{2}}{\sum_{i=1}^{n}{\left(x_{i}-\mu_{i}\right)}^{2}}$	1
RMSE	$RMSE=\sqrt{\frac{1}{n}\sum_{i=1}^{n}{\left(x_{i}-y_{i}\right)}^{2}}$	0
MAE	$MAE=\frac{1}{n}\sum_{i=1}^{n}\left|x_{i}-y_{i}\right|$	0
MAPE	MAPE=$\frac{1}{n}\sum_{i=1}^{n}\left|\frac{x_{i}-y_{i}}{x_{i}}\right|\times 100\%$	0

Note: $x_{i}$ denotes the observed value, $y_{i}$ the predicted value, $p_{i}$ the mean of the observed values, $\mu_{i}$ the mean of the predicted values, and n the total number of samples in the dataset.

**Table 3:** Statistical evaluation of the multiple models.

Dataset	Model	$R^{2}$	RMSE	MAE	MAPE
Training set	SVM	0.8531	2.5940	1.7703	24.9984
	XGB	0.9460	1.6125	1.1731	15.4347
Test set	SVM	0.8593	2.8182	2.1075	24.3626
	XGB	0.8934	2.2373	1.6802	17.8607

**Table 4:** Evaluation of different models for the ratio between predicted and actual values.

Model	Max	Min	Mean	Std	CV (%)
XGB	3.4848	0.3034	1.0524	0.2720	25.85
SVM	5.5094	−1.6181	1.0725	0.5248	48.93
Bhargava et al. [46]	5.1330	0.0032	0.9767	0.5678	58.13
Kivell [47]	5.8548	0.0339	1.1801	0.7216	61.15
Lee et al. [7]	6.0906	0.0877	1.0908	0.7122	65.29

**Table 5:** Performance evaluation metrics of different models.

Model	R^2^	RMSE	MAE	MAPE
XGB	0.9354	1.7388	1.2755	15.92
SVM	0.8543	2.6387	1.8375	24.87
Bhargava et al. [46]	0.7382	3.5444	2.7772	35.99
Kivell [47]	0.7475	3.4807	2.6275	38.87
Lee et al. [7]	0.7413	3.5233	2.6717	36.58

## Data Availability

The original contributions presented in this study are included in the article. Further inquiries can be directed to the corresponding author.
